# Beyond proof-of-concept: Validating robust automated diffuse lower-grade glioma segmentation for clinical applications in longitudinal follow-up

**DOI:** 10.1093/noajnl/vdaf256

**Published:** 2025-12-10

**Authors:** Jeremy Deverdun, Guillaume Clain, Margaux Verdier, Hugues Duffau, Liesjet E H Van Dokkum, Nicolas Menjot de Champfleur, Justine Meriadec, Mathilde Carriere, Amelie Darlix, Emmanuelle Le Bars

**Affiliations:** Department of Neuroradiology, Institute of Human Functional Imaging, Montpellier University Hospital; Department of Neuroradiology, Institute of Human Functional Imaging, Montpellier University Hospital; Department of Neuroradiology, Institute of Human Functional Imaging, Montpellier University Hospital; Department of Neurosurgery, Gui de Chauliac Hospital, Montpellier University Medical Center, Montpellier (H.D.); Team “Plasticity of Central Nervous System, Stem Cells and Glial Tumors”, INSERM U1191, Institute of Functional Genomics, Montpellier (H.D.); Department of Neuroradiology, Montpellier University Hospital; Department of Neuroradiology, Montpellier University Hospital; Department of Neuroradiology, Montpellier University Hospital; Department of Neuroradiology, Montpellier University Hospital; Department of Medical Oncology, Institut Régional du Cancer de Montpellier, University of Montpellier, Montpellier (A.D.); Institute of Functional Genomics IGF, University of Montpellier, CNRS, INSERM, Montpellier (A.D.); Department of Neuroradiology, Institute of Human Functional Imaging, Montpellier University Hospital; Department of Neuroradiology, Montpellier University Hospital

**Keywords:** automated segmentation, clinical MRI, diffuse lower-grade glioma, longitudinal follow-up, tumor volume

## Abstract

**Background:**

Monitoring diffuse lower-grade glioma (DLGG) evolution on MRI is challenging due to their infiltrative nature and post-surgical deformations. The RANO criteria recommend assessing the 2D tumor size, but volume analysis remains the gold standard for calculating growth rate. Manual tumor segmentation, however, is time-consuming, limiting its use in clinical practice. Automated segmentation tools like nnU-Net are promising but require clinical validation.

**Methods:**

We used 1971 MRI exams from 207 DLGG patients to train and validate an in house-developed nnU-Net al.orithm. The dataset included scans from various MRI systems, with 2D and 3D FLAIR and T1-weighted acquisitions, that were divided into derivation (*N* = 1771) and validation (*N* = 200) sets with matching 2D and 3D FLAIR ratios. The algorithm’s automated segmentations (AS) were compared with manual segmentations (MS) by expert neuroradiologists using the dice similarity coefficient (DSC) and Intersection over Union (IoU). Tumor volume and mean tumor diameter (MTD) were compared using Lin’s concordance correlation coefficient (CCC) and Bland-Altman tests.

**Results:**

The median nnU-Net DSC and IoU were 0.93 and 0.86, respectively. In the validation cohort, 64% of exams had excellent (≥0.9), 31% good ([0.7-0.9]), 3.5% unsatisfactory ([0.5-0.7]), and 1.5% poor DSC (<0.5). Higher DSC correlated with larger tumor volumes (*P* < .001). Tumor volume and MTD showed near-perfect concordance between AS and MS (CCC: 0.991 and 0.989, respectively).

**Conclusions:**

Our automated nnU-Net segmentation tool demonstrates high accuracy and potential for clinical integration, enhancing DLGG monitoring and management.

Key Points

- Automated nnU-Net segmentation achieves 0.93 Dice similarity for gliomas.
- Near-perfect concordance in tumor volume measurements: automated vs manual.
- Clinical-ready tool enhances longitudinal monitoring of diffuse gliomas.

Importance of the Study

This study validates an in house developed automated nnU-Net-based segmentation tool for diffuse lower-grade gliomas (DLGG), demonstrating high accuracy compared to manual segmentation (MS) by expert neuroradiologists. With a median Dice similarity coefficient of 0.93 and near-perfect concordance in tumor volume and mean tumor diameter measurements, the tool significantly enhances tumor monitoring. Unlike traditional 2D methods recommended by the RANO criteria, this volumetric approach answers to the challenges of glioma infiltrative growth and post-surgical remodeling. The tool’s ability to process longitudinal MRI data from diverse sources highlights its robustness and clinical applicability. By reducing the time and effort required for MS, this tool paves the way for widespread adoption of volumetric follow-up in routine clinical practice, ultimately improving patient management. Furthermore, it sets the stage for future advancements in integrating automated solutions into glioma research and care, bridging the gap between laboratory innovation and clinical translation.

Diffuse lower-grade gliomas (DLGG, diffuse grade 2 and 3 according to the WHO classification^1^) are rare tumors, with a yearly incidence of 1-2/10^5^. They typically affect young people in their third or fourth decade.^2^ DLGG are defined by a continuous growth, migrating along the white matter tracts over time and ending with an unavoidable malignant evolution resulting in a reduced life-expectancy. The prognosis is variable, with a median overall survival varying from 5 to over 15 years.^2^^,^^3^

Brain imaging is one of the key factors in the longitudinal follow-up of patients, as well as treatment planification and management. On conventional MRI, DLGG appear as a fairly homogeneous mass (hypersignal on T2 and FLAIR weighted sequences) with blurred borders, due to their infiltrative nature. Although contrast enhancement is classically associated with anaplastic transformation, it can also be observed in grade 2 and 3 DLGG.^4^ Thus, the best predictor of anaplastic evolution is the increase in the tumor expansion rate,^5^ requiring a precise monitoring of tumor growth and evolution. This is challenged by the tumor’s characteristics on the one hand (eg, slowly growing, infiltrating and blurred boundaries), and interventional effects of surgical resection (eg, tumor and brain remodeling with a surgical cavity) on the other. Longtime, subjective visual assessment of tumor size was used to evaluate its evolution. Yet, the human eye is unable to viably recognize small variations within tumors over time.^6^

Since 2012, experts emphasize the importance of quantitative measurements for DLGG follow-up.^7^ For example, the spontaneous growth rate, quantified by the evolution of measured mean tumor diameter (MTD),^8^ has been identified as an independent prognostic factor in grade 2 diffuse gliomas.^5^ This prognostic impact of MTD quantification has recently been confirmed in IDHmt 1p/19q codel DLGG.^9^ In line, the “Response Assessment in Neuro-Oncology” (RANO) criteria were introduced as standardized 2D assessment of tumor size by its length and width to evaluate treatment-response.^10^ Although functional in high-grade glioma with clear boundaries, their validity in case of DLGG is still debated.^11^ The use of a volumetric method to assess tumor progression or response to treatment might be more relevant in this setting.^12^^,^^13^ In the recently published update of the RANO criteria (RANO 2.0), the interest of volumetric measurements has been taken into account.^14^ However, manual segmentation (MS), although reproducible and precise^15^ is time-consuming, with a mean assessment time of 6 minutes per tumor in case of an expert observer on 2D FLAIR images. With 3D imaging, that is increasingly used in clinical practice, mean MS times are up to 37 minutes.^13^ Thus, methods enabling automated tumor segmentation and volume extraction have become primordial. Recent ­literature has corroborated nnU-Net’s performance for low-grade glioma segmentation,^16^ although its application was mainly restricted to preoperative data without cavities. In 2024, we published the encouraging data of a trained 2D nnU-Net al.orithm for DLGG segmentation over longitudinal follow-up with real life clinical data including pre- and post-surgery imaging with cavities, as well as imaging performed following chemotherapy and/or radiation therapy. However, although the algorithm performed well, the study was limited by a relatively small training set (*N* = 318 MRI) and few 3D acquisitions.^17^ In this work, our ultimate aim is to validate the algorithm for clinical application, by demonstrating its utility in routine clinical practice for automatic tumor volume estimation and longitudinal follow-up of patients with DLGG, by expanding our cohort and augmenting the percentage of 3D acquisitions.

## Methods

### Population

Participants were selected from the SPECIFY database (NCT04346472_UF9647, 2009-2023). SPECIFY was developed by the Montpellier University Hospital and includes imaging and clinical data of diagnosed DLGG patients, following the WHO 2016 classification.^18^ 207 patients were included with a longitudinal follow-up resulting in on average 9.55 ± 8.52 exams par patient, leading to the inclusion of a total of 1971 MRI exams. Reflecting clinical reality, these exams were acquired using multiple MRI systems and field strengths: 39% 3T (Siemens Healthcare, GE Healthcare, Philips Healthcare) and 61% 1.5T (Siemens Healthcare, GE Healthcare, Philips Healthcare, Canon) (Table 1). At the time of data collection, formal IRB approval was not required under French regulations. All participants gave informed consent and details of biological data are reported in Supplementary Material S1.

**Table 1.** Data description

Characteristic	Derivation set	Validation set	*P*-value
**MRI exams (*n*)**	1771	200	
**Demographics**			
**Gender (% (*n*))**			
Male	50.65% (897)	51% (103)	.82
Female	49.35% (874)	48.5% (97)	
**Age at first MRI (mean (±SD))**	42.29 (±9.97)	43.40 (±9.50)	.13
**MR field strength 3T (% (*n*))**	39% (692)	37.5% (75)	.66
**Machine vendors (% (*n*))**			.28
Canon	0% (0)	0% (0)	
Philips	0.6% (4)	0% (0)	
GE	1.4% (10)	0% (0)	
Siemens	98% (678)	100% (75)	
**MR field strength 1.5T (% (*n*))**	61% (1079)	62.5% (125)	.66
**Machine vendors (% (*n*))**			0.56
Canon	0.2% (2)	0% (0)	
Philips	14.3% (154)	16% (20)	
GE	24.1% (260)	20% (25)	
Siemens	61.4% (663)	64% (80)	
**MRI sequence**			
**T2 FLAIR (% (*n*))**			
2D (% (*n*))	87.58% (1551)	87.5% (175)	.97
3D (% (*n*))	12.42% (220)	12.5% (25)	
2D - Average resolution (xyz) (±SD)	3.03 (±1.52)	3.17 (±1.57)	.23
3D - Average resolution (xyz) (±SD)	0.7349 (±0.49)	0.764 (±0.57)	.78
**T1-weighted**			
Average resolution (xyz) (±SD)	2.74 (±1.62)	2.87 (±1.68)	.28

Notes. A total of 1971 MRI exams performed in 207 patients were included (9.55 ± 8.52 exams per patient). The cohort was split into a derivation (90%) and an external validation set (10%), with comparable 2D/3D T2FLAIR ratio. The derivation cohort was composed of 1771 MRI exams for automated segmentation algorithm training. To evaluate the trained algorithm an external validation cohort of 200 exams was used. Due to varying imaging resolutions, the average voxel size in mm^3^ was reported. To assess comparability between the 2 cohorts, *P*-values were calculated with a *χ*^2^-test for gender, magnetic field strength, machine vendors, and T2 FLAIR 2D/3D repartition. Student *t*-test were used for other variables. At *P* < 0.05, no significant differences were found between the cohorts, confirming the representativeness of the validation set in relation to the training set.

### Tumor Segmentations

Five experienced neuroradiologists with minimally 4 years of practice performed manual tumor segmentation (MS) using MRIcron software (NITRC: MRIcron: Tool/Resource Info. https://www.nitrc.org/projects/mricron) on FLAIR images with T1-weighed and follow up data when available. Of the 1971 MRI exams, 485 were previously fully manually segmented, with a mean segmentation time of 6 minutes for 2D and 37 minutes for 3D FLAIR images.^13^ To reduce the segmentation time, while ensuring segmentation quality, the remaining exams were first automatically segmented with the initial nnU-Net model,^17^ before being manually verified and corrected by one of the neuroradiologist. Correction times were 3.24 ± 5.4 min for 2D data and 5.54 ± 6.41 min for 3D. Finally, all 1971 MS tumor masks were re-evaluated and double-checked by 2 out of the 5 neuroradiologists. The resulting MS tumor masks were used as ground truth for both the training and the evaluation of the model.

### Model Training and Testing

As previously pointed out,^17^ 3D FLAIR acquisitions are slowly becoming the new standard in clinical practice, despite their longer MS times. Automatic segmentation software should thus be able to achieve good performance on both 2D and 3D acquisitions. Consequently, the exams were pseudo randomly split into a 90% derivation set (*N* = 1771) and a 10% external validation (*N* = 200) set that ensured a comparable 2D/3D ratio (87.5%/12.5%) in both sets. For the automated DLGG segmentation we used a nnU-Net specifically designed for segmenting biomedical data with an optimized and standardized pipeline. It considers data properties with a “dataset fingerprinting” step, and dynamically optimizes the CNN.^19^ We used the 2D and 3D-fullres architecture generated by the nnU-Net with its default parameters along with ensembling to estimate the best model for our dataset. Each training used a 5-fold cross-validation. Training was performed on a dedicated computer (GPU Nvidia RTX-4090 24Go vRAM, CPU Intel Core i9-13900KF, 128 Go DDR5).

Each MRI exam was composed of aT1-weigthed (post contrast if available) and T2 FLAIR imaging. Prior to training, the 3D T1-weighted images were co-registered to the T2 FLAIR images using affine transformation. Both were then used as input channels to the network, together with the corresponding MS. The trained network was then used to generate automated segmentations (AS).

In deep learning, often, the more data you have and the more various your data are, the better your model will be. To evaluate the model performance as a function of the number of training exams, we trained in parallel four different sized cohorts, composed of respectively 318, 1009, 1771, and 1971 exams. The first corresponds to our previous study.^20^ Model performance was compared between each cohort.

### Derived Metrics

For both AS and MS, tumor volumetric values (cm^3^) were extracted and plotted over time. Volumetric values were used to estimate the MTD (1).


(1) $$Truemeantumordiameter\left(mm\right)={\left(2*Volume\right)}^{\frac{1}{3}}*10$$

The MTD was used to calculate the velocity of diameter expansion (VDE (2)), in millimeters per year, for AS and MS, between two time points (time in years).


(2) $$\mathrm{Velocity}\;of\;\mathrm{Diameter}\;\mathrm{Expansion}\;(\frac{mm}{\mathrm{year}})=\frac{\Delta \mathrm{MTD}}{\Delta \mathrm{time}}$$

In our previous work, we highlighted VDE evolution of 6 “trial patients” with longitudinal follow-up. We repeated this analysis using the novel trained algorithm to compare its’ performance and impact on clinically relevant information. The VDE was calculated between 2 time-points that were minimally six months apart.^21^ When participants had an initial surgery, VDE was determined from the first exam post-surgery and in case of a chemotherapy, VDE was calculated between the first and the last MRI exam acquired during treatment period.

### Evaluation Metrics

The Dice Similarity Coefficient (DSC) and Intersection over Union (IoU) were calculated to assess the overlap between manual and AS. DSC emphasizes similarity, while IoU provides a clear measure of overall overlap. Segmentation performance was evaluated for both the derivation and the external validation sets.

The AS’ performance to assess tumor volume and MTD equivalently to the ground truth was evaluated by Lin’s concordance correlation coefficient (CCC)^22^ and a Bland-Altman test.^23^ These tests were also used to evaluate the correlation between the AS and the MS-based VDE for the trial patients of our previous work.^17^ Statistical significance was set at *P* < .05.

## Results

### Training and Validation Sets

While controlling for the 2D/3D ratio in the both sets (*P* = .97, Student *t*-test), the 2 cohorts exhibited comparable characteristics in terms of field strength (*P* = .66, *χ*^2^-test), machine vendors at 3T (*P* = .28, *χ*^2^-test) or 1.5T (*P* = .56, *χ*^2^-test), 2D (*P* = .23, Student *t*-test) and 3D (*P* = .78, Student *t*-test) T2-FLAIR voxel size, or T1-weighted voxel size (*P* = .28, Student *t*-test) (Table 1). The median manually segmented tumor volume was 21.65 cm^3^ (interquartile range IQR = 24.9) ranging from 0 to 375.34 cm^3^ (details in Supplementary Material S2).

### Model Performances: DSC and IoU

The median nnU-net DSC between MS and AS for both the derivation and external validation cohort was 0.93 (IQR: 0.09 and 0.12, respectively), with an IoU of 0.86 (IQR: 0.16 and 0.2). While 2D data showed a higher median DSC (0.93 (IQR:0.09)) compared to 3D data (0.91 (IQR:0.13)) in the derivation cohort, this trend was inverted in the external validation cohort (0.92 (IQR:0.12) versus 0.94 (IQR:0.12)) (Table 2).

**Table 2.** Dice Similarity Coefficient (DSC) and Intersection over Union (IoU) achieved on derivation and external validation cohort between MS and AS

	DSC (IQR)		IoU (IQR)	
	Derivation cohort	External validation cohort	Derivation cohort	External validation cohort
2D+3D	0.93 (0.09)	0.93 (0.12)	0.87 (0.16)	0.87 (0.20)
2D	0.93 (0.09)	0.92 (0.11)	0.87 (0.15)	0.86 (0.19)
3D	0.91 (0.13)	0.94 (0.12)	0.83 (0.21)	0.89 (0.21)

Notes. Median values along with interquartile range (IQR) are reported for all data, and 2D-only and 3D-only data. While 2D data have higher median DSC (0.93) compared to 3D (0.91) in the derivation cohort, this trend is inverted within the external validation cohort (0.92 vs 0.94).

Within the validation cohort (Figure 1), 64% of the exams had a very good DSC between MS and AS (≥0.9), 31% a good ([0.7 to 0.9]), 3.5% an unsatisfactory ([0.5 to 0.7]), and 1.5% had a poor DSC (<0.5) (Figure 1). Higher DSC scores were obtained for larger MS tumor volumes (*P* < .001, Kruskal–Wallis test). The mean tumor volumes were 35.68 cm^3^ (±32.25 cm^3^) for very good DSC, 22.51 cm^3^ (±21.08 cm^3^) for good DSC, 8.24 cm^3^ (±9.16 cm^3^) for unsatisfactory DSC and 1.47 cm^3^ (±0.66 cm^3^) for poor DSC. In contrast, neither the FLAIR 2D to 3D ratio (*P* = .63, *χ*^2^-test), the 1.5T to 3T ratio (*P* = .64, *χ*^2^-test), the 2D-voxel size (*P*  = .15, Kruskal–Wallis test), nor the 3D-voxel size (*P* = .86, Kruskal–Wallis test) were statistically different between DSC categories.

**Figure 1.** DSC repartition and characteristics in the validation cohort. DSC were classified as very good (≥0.9), good ([0.7-0.9]), unsatisfactory ([0.5-0.7]) and poor (<0.5). Illustrations of typical segmentations results per category are displayed with two slices per illustration, in green the MS, in red the AS and in yellow the overlap. 64% of the exams are classified as very good, 31% as good and only 5% as either unsatisfactory or poor. Segmentations with higher DSC tend to have higher volume. Proportion between categories were statistically compared using Chi-squared test, while continuous values were compared using Kruskal–Wallis test.

Examples of each DSC category are provided in Figure 1. Each case highlights the main difference between AS and MS. In these examples, while small tumor parts were clearly overlooked by AS for the very good DSC category, the good DSC category is more ambiguous with an hypersignal that might indeed be tumoral but was not manually segmented by the expert neuro-radiologists. Finally, as becomes clear with the example of a poor DSC case, when the tumor volume is low, a small absolute segmentation mistake in either AS or MS induces relatively large volume differences between volume estimations, leading to a poor DSC (Table 4).

### Model Performances: Derived Metrics

Regarding the derived metrics, almost perfect concordance between AS and MS was observed within the derivation cohort for the tumor volume (CCC: 0.997) and the MTD (CCC: 0.995). These results were confirmed within the validation cohort for volume and MTD (CCC: 0.991 and 0.989, respectively) with an average 0.9 cm^3^ and 0.5 mm underestimation of volume and MTD by AS ­compared to MS.

### Trial Patients

Figures 2 and 3 show respectively the newly automated extracted tumor volume evolution over follow-up, and the corresponding Bland-Altman correlation between AS and MS of these patients. To recall, the selected patients are the same as those presented in our previous study.^17^ Surgical intervention is indicated by a dashed line when present. Overall, the MS-based volume and MTD estimations tend to be slightly larger than those based on the AS with a mean difference of 0.9 cm^3^ and 0.6 mm respectively on the Bland-Altman plots (Figure 3). Both methods show almost perfect concordance for volume and MTD (CCC: 0.0993 and 0.990). In contrast, for Patient 2, a small discordance can be seen between points 3 to 5, with AS showing an increase while MS a decrease. The VDE estimated solely on the trial patients, also showed substantial concordance (CCC: 0.967) with a slight 0.1 mm/year overestimation of the AS compared to MS.

**Figure 2.** Evolution of the tumor volume over time in trial patients. For each patient, the variation of the tumor volume over time was plotted for the automated (AS, gray, empty dots) and manual segmentation (MS, black, full dots). In case the patient underwent a surgery (patient 2 to 6), the post-surgical exam is marked with a dashed line. Triangles indicate exams included in the validation cohort.

**Figure 3.** Bland-Altman of derived metrics in trial patients. Bland-Altman of derived metrics in trial patients. From left to right, the segmented volumes: MS and AS volumes showing a slight underestimation of the AS of 0.9 cm^3^, the corresponding MTD: a slight underestimation of the AS of 0.5 mm and the VDE calculations based on the segmented volumes: velocity of diameter expansion (VDE) showing a slight overestimation of the AS of 0.1 mm/year.

### Correlations between Model Performance and Number of Exams

AS performance as a function of the number of training exams included in the derivative cohort is reported in Figure 4. The DSC between AS and MS for 318, 1009, 1771 and 1971 exams were respectively 0.82 (IQR: 0.13), 0.89 (IQR: 0.11), 0.93 (IQR: 0.09), 0.93 (IQR: 0.09). A Wilcoxon rank-sum test confirmed that the increase in DSC with increasing derivative cohort size was significant between *N* = 318 and 1009 exams (*P* < .001), *N* = 1009 and 1771 (*P* < .001). However, no further significant increase was observed between *N* = 1771 and  1971 (*P* = .84).

**Figure 4.** Derivative cohort Dice Similarity Coefficient (DSC) according to the number of subjects in the derivation set. Estimated DSC (*N* = 318, 1009, 1771, and 1971) are plotted with circles and interquartile range by a light gray area. Statistical differences (*P* < 0.05) between points are displayed as stars (Wilcoxon rank-sum test). DSC increases until 1771 exams (0.82, 0.89, 0.93, and 0.93). No statistical difference was found between 1771 and 1971 exams (*P* = 0.84).

## Discussion

### Model Performances

Building upon our previous work that demonstrated the feasibility of an automated tumor segmentation with a nnU-Net for longitudinal DLGG follow-up, we now aimed to validate its utility in our clinical practice. Recall that correct volume estimation is essential to precisely monitor tumor progression or response to treatment in the follow-up of patients with DLGG.^7^^ ,^^12^ The volume is used to calculate the MTD, which over longitudinal follow-up is used to calculate the VDE. The VDE is currently the best indicator of sudden changes in DLGG growth that can be associated with anaplastic evolution.^8^ Although the MTD can be estimated sufficiently correct using 3D-linear measurements in a time-efficient manner, it remains an approximation.^13^

Thus, by expanding our dataset and by incorporating more 3D-FLAIR acquisitions, we aimed to both improve the model performance and to provide reliable estimates of tumor volume and the corresponding MTD for clinical use. Here, we demonstrated that our expanded model not only outperformed our previous one, as signified by a DSC increase from 0.82 to 0.93, but also that it outperformed models of comparable studies on automated tumor volume segmentation.^19^^,^^24–28^ Only one recent study^16^ achieved an equivalent DSC (0.93) in a small series of low grade gliomas, however, required more input images (FLAIR, T1, and T2-weigthed) and they used only pre-surgical images. The absence of surgical cavities facilitates segmentation and lowers the risk of false positives and negatives, but more importantly, it does not reflect the clinical reality especially in case of longitudinal follow-up. Our study design was focused on the validation of AS for both volume and MTD estimation regardless of therapeutic interventions that might have occurred during follow-up like chemotherapy, radiation therapy, or surgery. Having such a real-life clinical database increases the validity of the AS model for clinical practice.

Although on some occasions (see eg, Figure 1, Good DSC) it was difficult to identify who, between the AS and the MS, was right and who was wrong in case of small volume difference, the corresponding MTD estimation was almost perfect in the derivation cohort and substantial in the external validation cohort. Recognizing the importance of the VDE, we subsequently attempted to provide some insight into its validity in a subset of patients. Unfortunately, we were unable to explore the full patient database, as a proper evaluation of the automatic VDE estimation would have required a dedicated work, with regularly timed imaging follow-up. We focused on the same 6 patients that were evaluated as part of our previous work.^17^ First, it is important to keep in mind that the extended model differs from our feasibility model. The extended model is based on 2D/3D images with a pseudo random data distribution that honored comparable 2D/3D ratios between the training and external validation set, whereas the feasibility model was lacking of 3D T2FLAIR data, allowing full random data distribution. Nevertheless, we believe that illustrating the evolution of the model on the same data and figures is important to fully apprehend our model improvements, when dealing with deep learning approach. Secondly, the longitudinal data-set of one patient is composed of images that were part of the training set and of the external validation set. That said, the concordance between MS and AS of this specific subset was comparable to that of the overall data-set, and no outliers were observed as a function of dataset provenance (training vs external validity), confirming the representativity of our subset. Moreover, the VDE concordance was substantial between MS and AS. In fact, the AS and MS based VDE values appear much closer and more comparable, in line with the increased concordance, than in our previous paper. The average differences and standard deviations between AS and MS are also lower. This visually and statistically demonstrate the model improvements at any given instant during and over follow-up, while suggesting that the VDE can equally reliably be extracted with the current AS model and thus be used to evaluate patients’ clinical outcome in future clinical studies.

### Model Strengths

As pointed out by our previous work, the percentage of 2D T2FLAIR acquisitions will decrease over time due to the development of 3D acquisitions. The latter provide more precise information without increase in acquisition time,^29^^,^^30^ Our first model lacked 3D data which limited its generalizability. In our extended model we were able to double the number of 3D exams in the training and validation sets. A comparison between 2D and 3D data showed similar median DSC values, confirming the capacity of the extended model to segment tumor volume independent of the image dimension. This is encouraging as it suggests the model can handle both types of acquisitions effectively, offering flexibility for different clinical settings.

An important question in model building is the amount of input data. Does our current model have accumulated enough data, or could it be further improved by adding more data? To answer this question, we evaluated the model’s learning curve by exploring the impact of increasing the number of training exams on the AS performance. The results showed that the model performance improved with the increase in the number of training exams, until a plateau at 1771 exams. From this point on the median DSC did not further improve despite the inclusion of 200 additional exams. The presence of the plateau tends to highlight that our model is sufficiently trained on our daily heterogenous clinical data, that is characterized by not only both 2D and 3D imaging and cavities, but also by multiple constructors, field strengths and resolutions. Yet al.hough it has been optimized for our local practice, additional testing must be performed on data from different centers and countries to confirm its world-wide generalization.

While our previous work acted as a proof of concept, the current paper provides validation of AS for both 2D and 3D data in clinical routine with post-surgical data. Its high performance allows the automatic estimation of volume and MTD with a strong concordance to MS, while having a limited processing time (1 to 5 min for 3D T2FLAIR acquisition) making it relevant in a clinical environment. Even though corrections have occasionally to be performed manually, the segmentation time for the clinician is drastically reduced compared to full MS. Finally, having automatically extracted DLGG segmentation masks available, unlocks the possibility of intra-tumor parameter analysis and of assessing disease progression. For instance, its shape and location can be used for clinical research purposes, as well as for calculating associated 2D or 3D metrics such as tumor volume (V), width (W) and perpendicular tumor width (PW) used in the updated RANO2.0 criteria.^14^ The tool’s ability to provide rapid, reproducible volumetric measurements across large cohorts facilitates the systematic application of RANO 2.0 volumetric thresholds and opens new avenues for refining response assessment criteria. Additionally, automated segmentation enables advanced radiomic analysis of tissue characteristics, which has shown promise in distinguishing pseudo progression from true recurrence in gliomas, though dedicated post-radiotherapy datasets would be required for validation in lower-grade gliomas.

### Limitations

Several limitations still need to be addressed, particularly concerning the data used to train the model. Firstly, although we included different vendors, there is an overrepresentation of data from Siemens machines. While this reflects the actual distribution of 1.5T machines in France, the lack of data from other vendors at 3T could hinder the model’s generalizability for exams conducted on those systems. In terms of imaging data, although we have significantly increased the number of 3D T2FLAIR scans, they still only account for 12.4% of the overall dataset, despite the fact that all recently acquired data were in 3D. While we did not observe significant performance differences between 2D and 3D FLAIR (Figure 1), we believe that increasing the proportion of 3D exams is necessary to better align with current practices. Finally, the model’s segmentations still require visual inspection and, in some cases, manual correction to achieve accurate tumor delineation. For instance, the model is not designed to differentiate between tumoral and cicatricial FLAIR, which could lead to an overestimation of the tumor volume. Also, in case of small residual tumoral volumes at the edges of the cavity, differentiation between inflammation and tumor tissue is not unequivocal. A trained clinician’s eye remains thus essential and should always verify the segmented DLGG mask on the corresponding image.

## Conclusion

This newly trained automated segmentation tool was trained to segment DLGG in patients with various clinical characteristics and at different stages of the oncological history by using MRI exams with a great variety in terms of magnetic field strength, machine constructor, sequences acquisition (2D vs 3D) or resolution. Because of this data heterogeneity, that reflects the real-life clinical setting, the model can be used in clinical practice. The model provides, in a time-efficient manner, an accurate estimation of important metrics including tumor volume and MTD for both 2D and 3D FLAIR acquisitions. As the observed differences between the ground truth of MS and automatic estimations were mainly small, they are unlikely to impact patient management. Using our automated segmentation tool provides an important time-gain for clinicians, especially in case of 3D imaging. Still, the clinician’s eye is always required to verify the correctness of the segmentation, especially in the case of small(er) tumors. Next steps include model validation with data form various centers, and model integration into an environment that facilitates its access and use in everyday practice.

## Supplementary Material

vdaf256_Supplementary_Data

## Data Availability

The datasets generated and/or analyzed during the current study are not publicly available due to institutional and legal restrictions related to patient confidentiality. However, data may be made available from the corresponding author upon reasonable request and subject to appropriate ethical and legal approvals.
