# Supplementary material for: Beyond proof-of-concept: Validating robust automated diffuse lower-grade glioma segmentation for clinical applications in longitudinal follow-up
Source: Neurooncol Adv. 2025 Dec 10;8(1):vdaf256. doi: 10.1093/noajnl/vdaf256 (PMC12901730; doi:10.1093/noajnl/vdaf256)
Supplement: vdaf256_Supplementary_Data [file vdaf256_supplementary_data.docx]

## Supplementary material

### Biological data (OMS 2016)

| IDH Status | 1p/19q Codeletion | N |
| --- | --- | --- |
| Mutated (1m [92.5%], 2m [7.5%]) | True | 654 |
| Mutated (1m [97.8%], 2m [2.2%]) | False | 1164 |
| Mutated (1m [100%], 2m [0%]) | Unknown | 16 |
| Wild type | - | 99 |
| Unknown | False | 10 |
| Unknown | True | 27 |
| Unknown | Unknown | 1 |

### Distribution of MS tumoral volumes


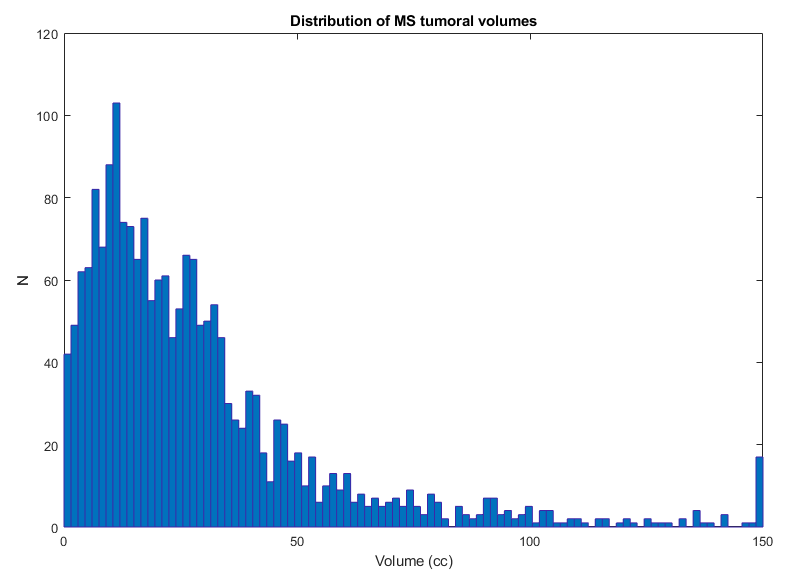


### DSC versus MS volume

Volume are represented in cubic centimer (cm^3^). Lowest DSC (Dice Similarity Coefficient) is achieved with small tumoral volume.

### Bland altman plots in validation cohort for volume and MTD


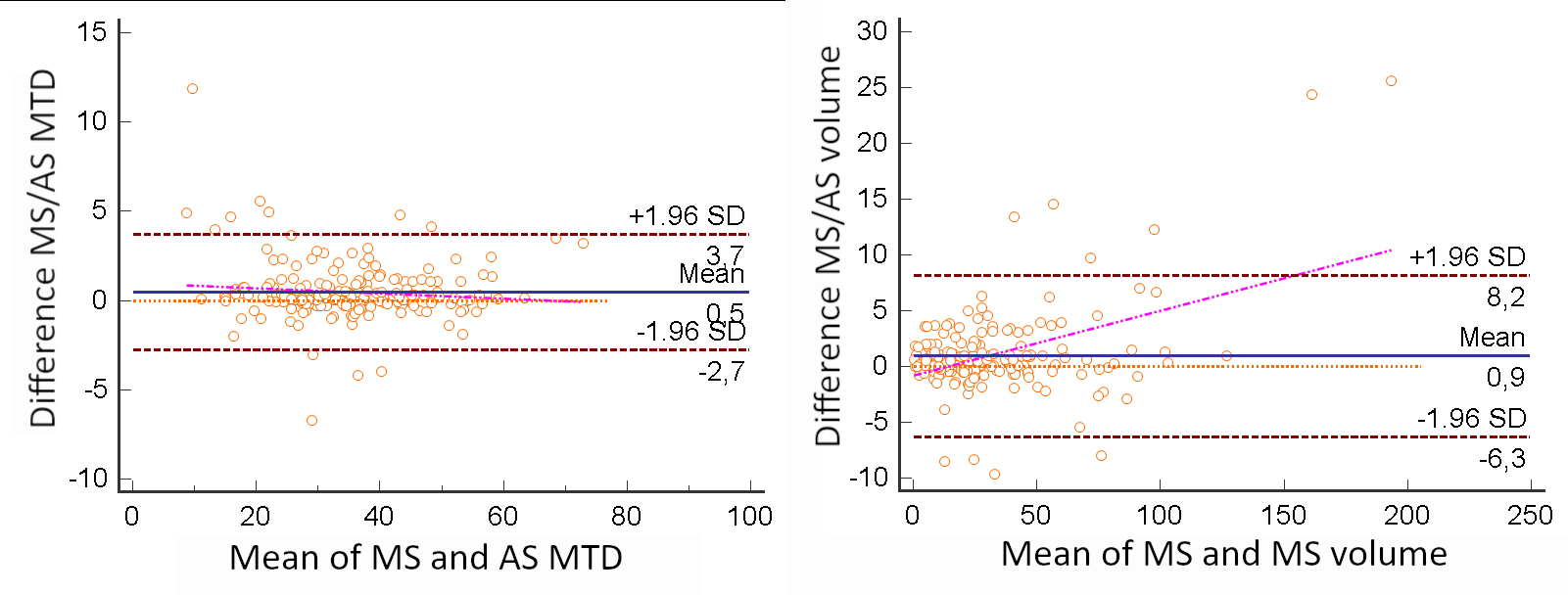


Bland-Altman plots in validation cohort for volume and MTD. On the left, MTD in validation cohort: AS underestimate the MTD of 0.5 mm. On the right, segmented volume in validation cohort: Manual segmentation (MS) and automated segmentation (AS) volumes showing a 0.9 cm^3^ underestimation with the AS.

### MTD and VDE on trial patients


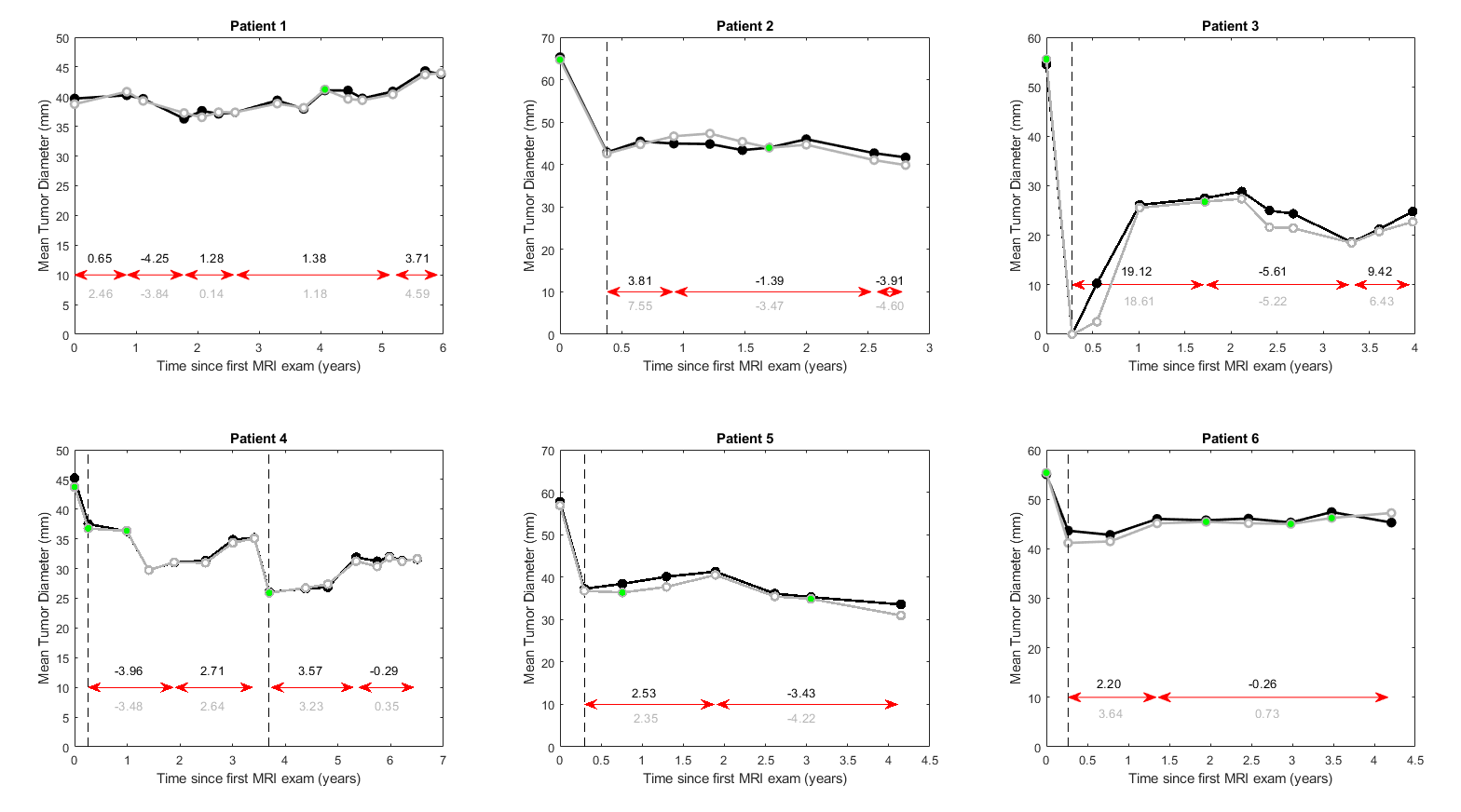


Mean tumor diameter (mm) for automated (AS, gray, empty dots) and manual segmentation (MS, black, full dots) over the years, the Velocity of diameter expansion (VDE) is calculated between specific time points. The VDE describes the tumor diameter evolution between two MRI exams and evaluate the tumor evolution along the follow-up, to evaluate the need of a treatment (surgery, chemo or radiotherapy) or its efficiency. Periods of chemotherapy treatment are highlighted in gray blocs. In case there was a surgery, the post-surgical exam is marked with a dashed line. In case of an initial surgery (patient 2 to 6), the VDE is calculated starting from the post-surgery MRI exam (A). In case there was a surgery during the follow-up, the VDE is calculated until the pre-surgery exam (patient 4, C) and the next VDE calculation starts from the post-surgery exam (patient 4, D). The exams included the validation cohort are highlighted in green.
